# Epidemiological characteristics of New Delhi Metallo-β-Lactamase-producing *Enterobacteriaceae* in the Fourth hospital of Hebei Medical University

**DOI:** 10.1186/s12879-023-08242-8

**Published:** 2023-05-05

**Authors:** Mengsi Zhao, Jing He, Ran Zhang, Junhua Feng, Yanli Deng, Jinyan Zhang

**Affiliations:** Department of Clinical Laboratory, The Fourth Hospital of Hebei Medical University, Shijiazhuang, 050000 China

**Keywords:** Carbapenem-resistant *Enterobacteriaceae*, New Delhi Metallo-β-lactamase, Clinical characteristics, Multilocus sequence typing

## Abstract

The epidemiological characteristics of New Delhi Metallo-β-Lactamase-Producing (NDM) *Enterobacteriaceae* were analyzed to provide theoretical support for clarifying the distribution characteristics of carbapenem-resistant *Enterobacteriaceae* (CRE) in the hospital environment and early identification of susceptible patients. From January 2017 to December 2021,42 strains of NDM-producing *Enterobacteriaceae* were gathered from the Fourth Hospital of Hebei Medical University, primarily *Escherichia coli*, *Klebsiella pneumoniae*, and *Enterobacter cloacae*. The micro broth dilution method combined with the Kirby-Bauer method was used to determine the minimal inhibitory concentrations (MICs) of antibiotics. The carbapenem phenotype was detected by the modified carbapenem inactivation method (mCIM) and EDTA carbapenem inactivation method (eCIM). Carbapenem genotypes were detected by colloidal gold immunochromatography and real-time fluorescence PCR. The results of antimicrobial susceptibility testing showed that all NDM-producing *Enterobacteriaceae* were multiple antibiotic resistant, but the sensitivity rate to amikacin was high. Invasive surgery prior to culture, the use of excessive amounts of different antibiotics, the use of glucocorticoids, and ICU hospitalization were clinical characteristics of NDM-producing *Enterobacteriaceae* infection. Molecular typing of NDM-producing *Escherichia coli* and *Klebsiella pneumoniae* was carried out by Multilocus Sequence Typing (MLST), and the phylogenetic trees were constructed. Eight sequence types (STs) and two NDM variants were detected in 11 strains of *Klebsiella pneumoniae*, primarily ST17, and NDM-1. A total of 8 STs and 4 NDM variants were detected in 16 strains of *Escherichia coli*, mainly ST410, ST167, and NDM-5. For high-risk patients who have CRE infection, CRE screening should be done as soon as feasible to adopt prompt and efficient intervention measures to prevent outbreaks in the hospital.

**Supplementary Information:**

The online version contains supplementary material available at 10.1186/s12879-023-08242-8.

## Introduction

With the widespread use of carbapenems in clinical practice, carbapenem-resistant *Enterobacteriaceae* (CRE) is increasing [1]. In recent years, CRE has been reported in different countries and regions. Numerous investigations have demonstrated that the majority of CRE strains produce carbapenemases, the most prevalent carbapenemase genes were KPC and NDM [2–5]. Since the discovery of *bla*_NDM−1_ in *Escherichia coli* isolated from India in 2008, NDM-producing *Enterobacteriaceae* have been widely reported throughout the world, and Asia is thought to be the primary epidemic location. Carbapenem-resistant *Klebsiella pneumoniae* (CRKP) and carbapenem-resistant *Escherichia coli* (CREC) are the main types of *Enterobacteriaceae* [4–6].

The resistance rates of *Enterobacteriaceae* to imipenem and meropenem in China have climbed year over year and have now increased to nearly 10%, according to data from the China Antimicrobial Surveillance Network (CHINET). In this study, multiple methods were used to detect carbapenemases, and NDM-producing *Enterobacteriaceae* were screened out in 5 years. Through retrospective analysis, the distribution characteristics of NDM-type carbapenemases in the Fourth Hospital of Hebei Medical University were clarified, to monitor the development trend of carbapenemases, grasp its epidemic status, and promote the smooth implementation of hospital infection control.

## Methods

### Strain source

This study screened hospitalized patients in the Fourth Hospital of Hebei Medical University from January 2017 to December 2021. A total of 12,373 patients with bacterial infections were collected, 8725 patients were infected with Gram-negative bacteria, and 4987 patients were infected with *Enterobacteriaceae*. CRE infections (130) accounted for 1.05% of bacterial infections, and 42 CRE strains were NDM-producing *Enterobacteriaceae.*

### Strain identification

Strain identification of 42 strains of NDM-producing *Enterobacteriaceae* using matrix-assisted laser desorption time-of-flight mass spectrometry (bioMérieux, France). Calibration strain: *Escherichia coli* ATCC8739. Quality control strain: *Enterobacter aerogenes* ATCC1915.

### Antimicrobial susceptibility testing

The antimicrobial susceptibility testing was performed on the experimental strains using an automated VITEK^®^2 Compact system (bioMérieux, France) with *Escherichia coli* ATCC 25922 and *Pseudomonas aeruginosa* ATCC27853 as the quality control strains, and the results were determined according to the CLSI M100-S32 standard of 2022.

### Carbapenemase phenotypic and genotypic assays

The carbapenemase phenotype was detected using the modified carbapenem inactivation method (mCIM) in conjunction with the EDTA carbapenem inactivation method (eCIM); the genotypes of the carbapenemase were determined using the colloidal gold immunochromatography kit (Changsha Zhongjie Biotechnology Co., Ltd.) and real-time fluorescence PCR kit (Cepheid, USA), and the consistency of the two detection methods was verified.

### Information collection from medical records

The medical records of patients infected with NDM-producing *Enterobacteriaceae* were checked, and the following observations were recorded and counted: patients’ hospitalization department, gender, age, underlying disease, invasive operation before culture, history of antibiotics use, and hospitalization time.

### MLST and evolutionary analysis

The whole genome shotgun (WGS) strategy was used to sequence 11 strains of *Klebsiella pneumoniae* and 16 strains of *Escherichia coli* based on the Illumina NovaSeq sequencing platform to obtain bacterial genome sequences; MLST 2.0 (https://cge.cbs.dtu.dk/services/MLST/ ) for multilocus sequence typing, detecting the STs of the strains and obtaining the housekeeping gene sequences of the strains; using MEGA 11 (https://www.megasoftware.net) software for multiple sequence alignment and phylogenetic tree construction, and using iTOL online beautification tool (https://itol.embl.de) for Visualization.

### Accession number

The complete genome sequences of *Klebsiella pneumoniae* and *Escherichia coli* are deposited in the GenBank database as the accession number PRJNA903849 and PRJNA903692.

## Results

### Strain distribution

**Fig. 1:**
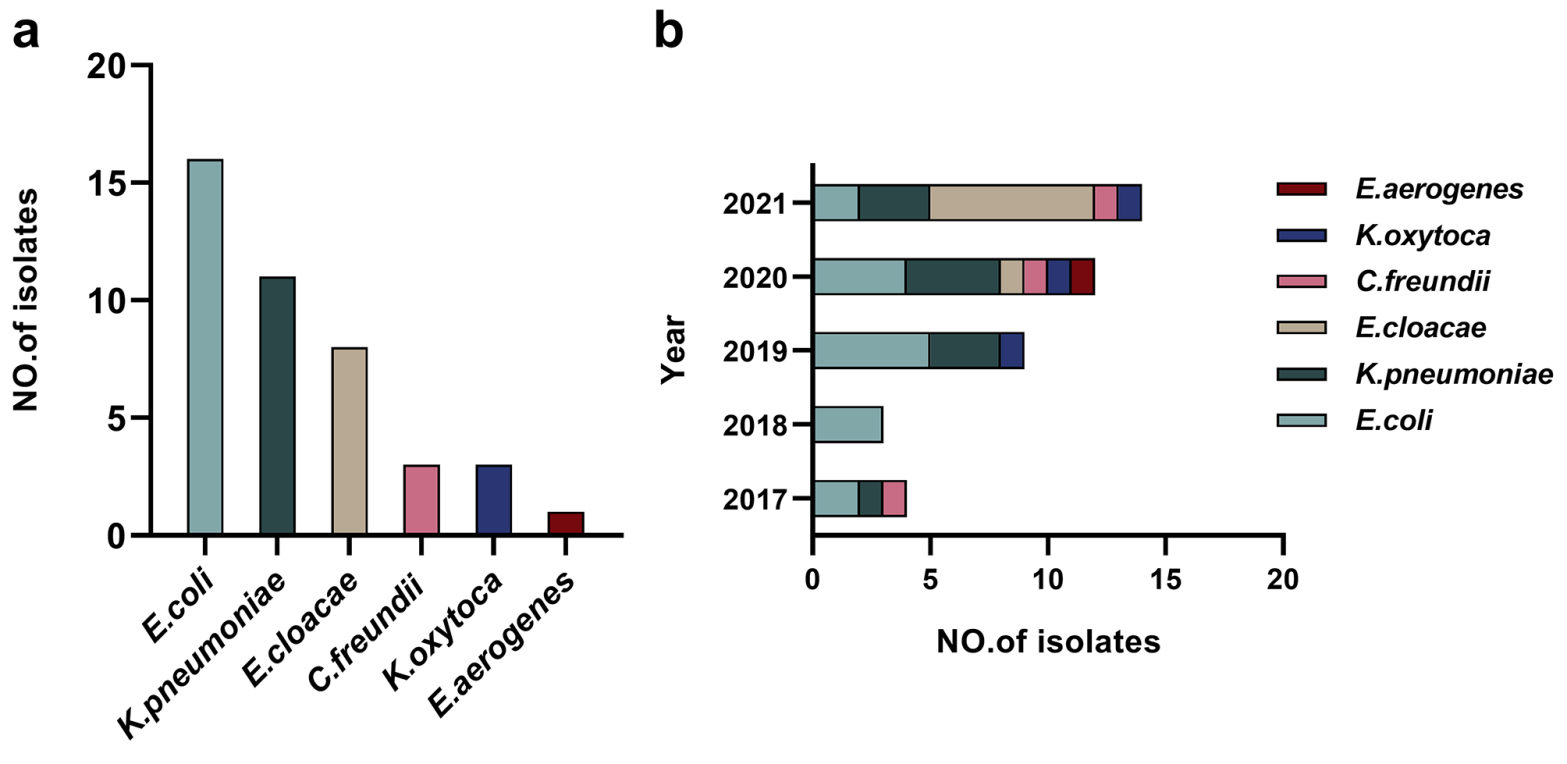
Type distribution and epidemic trend of NDM-producing *Enterobacteriaceae* a. Type distribution of NDM-producing *Enterobacteriaceae* b. The epidemic trend of NDM-producing *Enterobacteriaceae* from 2017 to 2021

Forty-two strains of NDM-producing *Enterobacteriaceae* in total, including *Escherichia coli* (16, 38.1%), *Klebsiella pneumoniae* (11, 26.2%), *Enterobacter cloacae* (8, 19.1%), *Citropebacterium freundii* (3, 7.1%), *Klebsiella oxytoca* (3, 7.1%), and *Enterobacter aerogenes* (1, 2.4%), were gathered for this research. Figure 1 displays the annual changes in the proportion of NDM-producing *Enterobacteriaceae* in our hospital since 2017.

### Antimicrobial susceptibility testing

The antimicrobial susceptibility testing results of the experimental strains are shown in Table 1. All NDM-producing *Enterobacteriaceae* showed multiple antibiotic resistance, highly resistant to meropenem and imipenem, with very low sensitivity to cephalosporins and penicillins and high sensitivity to amikacin, among which the sensitivity rates of *Klebsiella pneumoniae* and *Enterobacter cloacae* reached 100%.

**Table 1:** Antimicrobial susceptibility testing results of NDM-producing *Enterobacteriaceae*

	% (no. of susceptible isolates/no. of isolates tested)				
	By site		By organism		
**Antimicrobial agent**	**Isolated from sterile** **site (n = 28)**	**Isolated from nonsterile** **site(n = 14)**	***Escherichia coli*** **(n = 16)**	***Klebsiella pneumoniae*** **(n = 11)**	***Enterobacter cloacae*** **(n = 8)**
**Aztreonam**	17.9(5/28)	35.7(5/14)	6.3(1/16)	36.4(4/11)	37.5(3/8)
**Meropenem**	0(0/28)	0(0/14)	0(0/16)	0(0/11)	0(0/8)
**Imipenem**	0(0/28)	0(0/14)	0(0/16)	0(0/11)	0(0/8)
**Ceftazidime**	3.6(1/28)	0(0/14)	6.3(1/16)	0(0/11)	0(0/8)
**Cefatriaxone**	0(0/28)	0(0/14)	0(0/16)	0(0/11)	0(0/8)
**Cefepime**	3.6(1/28)	0(0/14)	6.3(1/16)	0(0/11)	0(0/8)
**Piperacillin**	0(0/28)	0(0/14)	0(0/16)	0(0/11)	0(0/8)
**Piperacillin/tazobactam**	3.6(1/28)	0(0/14)	0(0/16)	0(0/11)	0(0/8)
**Ciprofloxacin**	14.3(4/28)	35.7(5/14)	6.3(1/16)	18.2(2/11)	62.5(5/8)
**Levofloxacin**	11.1(3/27)	35.7(5/14)	6.7(1/15)	36.4(4/11)	50(4/8)
**Gentamicin**	21.4(6/28)	28.6(4/14)	18.8(3/16)	18.2(2/11)	12.5(1/8)
**Tobramycin**	14.3(4/28)	28.6(4/14)	12.5(2/16)	27.3(3/11)	12.5(1/8)
**Amikacin**	85.7(24/28)	92.9(13/14)	68.8(11/16)	100(11/11)	100(8/8)

### Antibiotics-resistant phenotypic screening and genotype testing

It was verified that all 42 strains collected in this study were metallo-carbapenemase-producing *Enterobacteriaceae*; the results of CRE genotype detection by colloidal gold immunochromatography and real-time fluorescence PCR showed high consistency, and the antibiotics-resistant genotypes of all 42 CRE strains were NDM.

### Bioinformatics analysis

Eleven strains of NDM-producing *Klebsiella pneumoniae* and 16 strains of NDM-producing *Escherichia coli* were analyzed by the ResFinder database (Additional file 2). It was found that all strains had multiple antibiotic resistance genes, and all carried carbapenem resistance genes. The majority of strains carried extended-spectrum β-lactamases (*bla*_CTX_, *bla*_SHV_, *bla*_TEM_, etc.), and other antibiotic resistance genes such as sulfonamides (*sul1*, *sul2*, *sul3*, etc.) and aminoglycosides (*aac(6’)-Ib-cr*, *aph(3’)-Ia*, etc.).

### Clinical characteristics of patients infected with CRE

There were 42 patients altogether, 28 of whom were men. 78.6% of those infected with CRE were over the age of 50, 35.7% had undergone surgery within one month. More than half of the patients had malignant tumors (23/42, 54.8%), and most of them had combined underlying diseases such as cardiovascular and cerebrovascular diseases, pulmonary diseases, hypertension, and a history of smoking. More than 50% of infected patients had used indwelling devices such as arteriovenous catheters and drainage tubes. 57.1% of CRE-infected patients used three or more antibiotics, and most patients used antibiotics for more than 14 days. In addition, nearly half of the patients with CRE infection (20/42, 47.6%) had ICU hospitalization experience. The results are shown in Table 2.

**Table 2:** Clinical characteristics of patients with NDM-Producing *Enterobacteriaceae*

Characteristic	Data
Female	14/42 (33.3)
Age	
13–17	3/42 (7.1)
18–49	6/42 (14.3)
50–64	14/42 (33.3)
65–79	13/42 (31.0)
≥ 80	6/42 (14.3)
**Underlying conditions**	
Pulmonary diseases	11/42 (26.2)
Cardiovascular disease	13/42 (31.0)
Kidney disease	6/42 (14.3)
Cerebrovascular disease	10/42 (23.8)
Cancer	23/42 (54.8)
Bacteremia/Sepsis	2/42 (4.8)
Surgery within a month	15/42 (35.7)
Diabetes mellitus	3/42 (7.1)
Hypertension	13/42 (31.0)
Immunodeficiency	1/42 (2.4)
Smoking history	17/42 (40.5)
**Indwelling devices prior to culture**	
Arterial cannula	25/42 (59.5)
Central venous catheter	35/42 (83.3)
Gastric tube	14/42 (33.3)
Tracheal cannula	20/42 (47.6)
Drainage tube	23/42 (54.8)
Urinary catheter	19/42 (45.2)
**Antimicrobial use prior to culture within 30 days**	
Cephalosporins	25/42 (59.5)
Carbapenems	17/42 (40.5)
Quinolones	10/42 (23.8)
Aminoglycosides	9/42 (21.4)
Penicillins	17/42 (40.5)
Glycopeptides	11/42 (26.2)
Sulfonamides	2/42 (4.8)
Monocyclic β lactams	2/42 (4.8)
Linezolid	8/42 (19.0)
Tigecycline	6/42 (14.3)
**Antimicrobial use ≥ 14 days**	26/42 (62.0)
**Antibiotics ≥ 3 types**	24/42 (57.1)
**Type of infection**	
Bloodstream infection	16/42 (38.1)
Abdominal infection	12/42 (28.6)
Lower respiratory tract infection	9/42 (21.4)
Urinary tract infection	6/42 (14.3)
Wound infection	3/42 (7.1)
**Glucocorticoids**	19/42 (45.2)
**ICU stay**	20/42 (47.6)

### Sequence typing and phylogenetic analysis

**Fig. 2:**
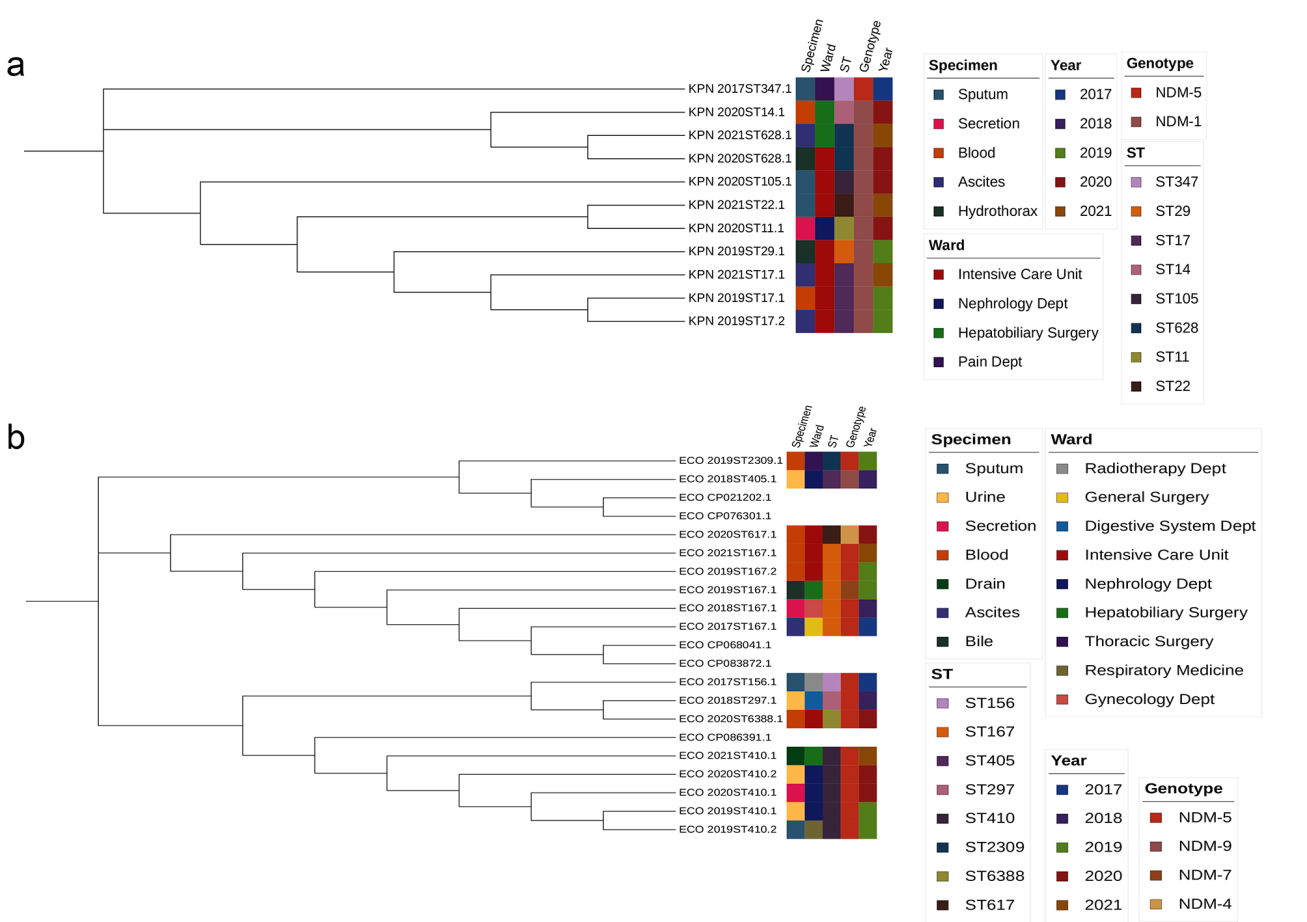
Phylogenetic analysis of NDM-producing *Klebsiella pneumoniae* and *Escherichia coli*.Annotated information includes the specimen type, ward, MLST typing, CRE genotype, and the year of the strains a. Phylogenetic analysis of NDM producing *Klebsiella pneumoniae* b. Phylogenetic analysis of NDM-producing *Escherichia coli*

In this study, CRKP was mainly divided into three evolutionary clusters, and CREC was divided into four different evolutionary clusters, as shown in Fig. 2. Multilocus sequence typing (MLST) showed that 11 strains of *Klebsiella pneumoniae* had 8 STs, mainly ST17 strains, with 3 strains detected; the genotypes of NDM-producing *Klebsiella pneumoniae* were NDM-1 and NDM-5, of which 10 strains were NDM-1. A total of 8 STs were found in 16 *Escherichia coli* strains, with ST410 and ST167 strains having the most (5 strains each); NDM-producing *Escherichia coli* genotypes comprised NDM-4, NDM-5, NDM-7, and NDM-9, with NDM-5 being the most common.

## Discussion

Data from the China Antimicrobial Surveillance Network showed that among the metallocarbapenemases (MBLs) clinically isolated from general hospitals in China in 2020, the NDM type was the most found, followed by the IMP type, and the VIM type was the least discovered. In this investigation, we discovered that 42 instances of metallocarbapenemase-producing *Enterobacteriaceae* collected in our hospital from January 2017 to December 2021 were of the NDM type, which is comparable with the prevalent status in China. We also discovered that since 2017, the prevalence of NDM carbapenemases has progressively increased in our hospital, and the range of strains has extended, similar to those reported in other Asian countries [7–9]. Compared with other carbapenemases, *bla*_NDM_ transmission has broader epidemiological characteristics, including (1) a wide range of Gram-negative hosts; (2) *Escherichia coli* and *Klebsiella pneumoniae* are used as conditional pathogens in the intestinal flora and can lead to patient infection through flora displacement; (3) the high population density in Southeast Asia, where it is widespread, makes it easy to disseminate antibiotics-resistant genes; (4) other resistance genes are often present on plasmids carrying *bla*_NDM_ [10].

According to the results of the antimicrobial susceptibility testing, *Enterobacteriaceae* carrying *bla*_NDM_ gene were highly resistant to most β-lactams and were sensitive to quinolones and aminoglycosides, especially amikacin. The vast majority of NDM-producing *Enterobacteriaceae* were sensitive to amikacin. This is consistent with the results of the above bioinformatics analysis, indicating that the coexistence of multiple antibiotic resistance genes gives the host bacteria multiple antibiotic resistance. It has been shown that the antibiotic resistance of clinical isolates to cephalosporins and aminoglycosides in developing countries, including China, is higher than that in developed countries [11]. Therefore, clinical attention should be paid to standardizing the principles and systems for the use of antimicrobial agents, and scientifically and rationally adjusting the dosing regimen.

In the present study, most of the patients with CRE infection were male, and the majority of elderly males were over 50 years old, accounting for 59.5%. As shown in Table 2, elderly patients, hypertension, diabetes, ICU stay, pulmonary disease, and various invasive operations were important clinical characteristics for CRE infection, which is generally consistent with the previous study by Pang F et al. [12]. The results of this study showed that 83% of patients received antibiotic therapy within 30 days prior to a positive culture, and more than half of these patients had a history of receiving more than three or using more than 14 days of antibiotics, 25 (25/42, 59.5%) patients received 3rd or 4th generation cephalosporins, and 17 (17/42, 40.5%) patients received carbapenems or penicillins. Overexposure to multiple antibiotics and a history of 3rd or 4th generation cephalosporins and carbapenems prior to infection explain the high rate of CRE infections [13, 14].

MLST analysis revealed the genetic diversity of CRE strains. It was shown that NDM-producing *Klebsiella pneumoniae* are distributed among numerous STs, and ST11, ST14, ST15, and ST147 are relatively common types that have been identified in several countries [15], although the current evidence is insufficient to prove that ST11, ST14, ST15, and ST147 are the prevalent clonal lineages mediating the spread of *bla*_NDM_ internationally, their distribution in several countries warrants further study for their distribution. The 11 NDM-producing *Klebsiella pneumoniae* strains in this study include 8 STs,2 STs (ST11 and ST14) are consistent with it, and the other six are less common and have some local specificity. Twenty-four variants of NDM have been identified so far, among which NDM-1 has the broadest host spectrum, with *Klebsiella pneumoniae* and *Escherichia coli* being the main carriers of *bla*_NDM−1_ [16], and NDM-1 was likewise the major NDM variant of *Klebsiella pneumoniae* in this study.

The 16 NDM-producing *Escherichia coli* strains in this study included 8 STs, mainly ST410 and ST167, with 5 strains detected each. Compared with other ST strains, the affinity between ST167 and ST617 is very close, with ST167 strains being the high-risk epidemic type in China [17, 18], and four of the five ST167 strains were NDM-5 variants, suggesting that ST167 is closely related to *bla*_NDM−5_ to some extent, which is consistent with previous studies [19, 20]. Additionally, even though 5 ST167 strain cases were found in this study, they were all from different wards and different years, preventing nosocomial infection outbreaks. In contrast, 2 ST410 strain cases were found in the nephrology department in June 2020 and were both NDM-5 variants, suggesting the possibility of nosocomial local transmission of CREC. NDM-5 was discovered in 2011 in a strain of *Escherichia coli* in the United Kingdom [21], and it differs from NDM-1 by having higher carbapenemase activity and amino acid changes at positions 88 (ValLeu) and 154 (MetLeu). Since then, strains carrying *bla*_NDM−5_ have been reported in numerous countries including Singapore, India, Japan, China, Australia, and the United States, and NDM-5 has become the second most reported NDM variant after NDM-1 in the world [22–24]. Notably, in previous reports, NDM-1 was likewise the most prevalent NDM variant among CRE strains in China, but our study showed that NDM-5 was predominant among CREC strains and accounted for 81.25%, and a similar situation was found in a teaching hospital in Chongqing [20], which reveals that CREC carrying *bla*_NDM−5_ is gradually becoming a new potential threat, and there is an urgent need for clinical attention and effective interventions to control its further spread.

## Conclusion

In conclusion, we should expand research on the molecular epidemiology of CRE and the prevalence trend of New Delhi Metallo-β-lactamase-producing *Enterobacteriaceae* to offer a scientific foundation for early detection of patients with high-risk CRE infection, early screening of patients with high-risk CRE infection, and prompt and effective implementation of therapies to improve patients’ clinical outcomes.

## Electronic supplementary material

Below is the link to the electronic supplementary material.

Supplementary Material 1

Supplementary Material 2

## Data Availability

The datasets that support the findings of this study are available in the NCBI GenBank database, https://www.ncbi.nlm.nih.gov/bioproject/. Accession codes are PRJNA903849 and PRJNA903692.
