# Supplementary material for: Epidemiological characteristics of New Delhi Metallo-β-Lactamase-producing Enterobacteriaceae in the Fourth hospital of Hebei Medical University
Source: BMC Infect Dis. 2023 May 5;23:298. doi: 10.1186/s12879-023-08242-8 (PMC10163796; doi:10.1186/s12879-023-08242-8)
Supplement: Supplementary file 2 — Supplementary Material 2 [file 12879_2023_8242_MOESM2_ESM.pdf]

Antibiotic resistance genes carried by NDM-producing *Enterobacteriaceae*

Strain	Carbapenems	β-lactams	Macrolides	Quinolones	Tetracycline	Fosfomycin	Sulfonamides	Aminoglycosides	Amphenicols	Trimethoprim	Rifampicin	Polymyxin
KPN_2017 ST347.1	*bla*$_{NDM-5}$	*bla*$_{TEM-176}$		*OqxA*、*OqxB*、*qnrS1*		*fosA*		*aph(3')-Ia*		*dfrA14*		
KPN_2019 ST29.1	*bla*$_{NDM-1}$	*bla*$_{TEM-1B}$、*bla*$_{SHV-187}$		*OqxA*、*OqxB*		*fosA*	*sul1*	*aac(3)-IId*、*aadA2*		*dfrA12*		
KPN_2019 ST17.1	*bla*$_{NDM-1}$	*bla*$_{SHV-94}$、*bla*$_{TEM-1B}$、*bla*$_{CTX-M-14}$、*bla*$_{LAP-2}$、*bla*$_{SFO-1}$	*mph(A)*	*OqxA*、*OqxB*、*aac(6')-Ib-cr*、*qnrB6*、*qnrS1*	*tet(A)*、*tet(D)*	*fosA*	*sul1*、*sul2*	*aac(3)-IId*、*aac(6')-Ib-cr*、*aph(6)-Id*、*aadA2*、*aadA16*、*aph(3'')-Ib*	*floR*	*dfrA1*、*dfrA12*、*dfrA27*	*ARR-3*	
KPN_2019 ST17.2	*bla*$_{NDM-1}$	*bla*$_{SHV-96}$、*bla*$_{SHV-94}$、*bla*$_{SHV-172}$、*bla*$_{CTX-M-14}$		*OqxA*、*OqxB*、*qnrS1*	*tet(A)*	*fosA3*	*sul1*	*aac(3)-IId*		*dfrA1*		
KPN_2020 ST14.1	*bla*$_{NDM-1}$、*bla*$_{OXA-1}$	*bla*$_{CTX-M-3}$、*bla*$_{SHV-28}$、*bla*$_{SHV-106}$		*OqxA*、*OqxB*、*qnrS1*、*aac(6')-Ib-cr*	*tet(A)*	*fosA*、*fosA3*	*sul1*	*aadA16*、*aadA1*、*aadA2b*、*aph(3')-VI*、*aac(6')-Ib-cr*、*aac(3)-IIa*	*catB3*	*dfrA1*、*dfrA27*	*ARR-3*	

Strain	Carbapenems	β-lactams	Macrolides	Quinolones	Tetracycline	Fosfomycin	Sulfonamides	Aminoglycosides	Amphenicols	Trimethoprim	Rifampicin	Polymyxin
KPN_2020 ST105.1	*bla*$_{NDM-1}$	*bla*$_{TEM-1B}$、*bla*$_{SFO-1}$、*bla*$_{SHV-11}$、*bla*$_{SHV-13}$、*bla*$_{SHV-70}$	*mph(A)*	*OqxA*、*OqxB*		*fosA*	*sul1*	*aadA2*、*aac(3)-IId*		*dfrA12*		
KPN_2020 ST628.1	*bla*$_{NDM-1}$、*bla*$_{OXA-1}$	*bla*$_{CTX-M-14}$、*bla*$_{LAP-2}$、*bla*$_{SHV-194}$、*bla*$_{SHV-26}$		*OqxA*、*OqxB*、*qnrS1*、*aac(6')-Ib-cr*	*tet(A)*	*fosA*、*fosA7*	*sul1*、*sul2*	*aac(3)-IId*、*aac(6')-Ib-cr*	*catB3*	*dfrA1*	*ARR-3*	
KPN_2020 ST11.1	*bla*$_{NDM-1}$	*bla*$_{SHV-182}$	*mph(A)*	*OqxA*、*OqxB*、*aac(6')-Ib-cr*、*qnrB52*	*tet(A)*	*fosA*	*sul1*、*sul2*	*aph(6)-Id*、*aadA2b*、*aph(3')-Ia*、*aadA16*、*aac(6')-Ib-cr*、*aph(3'')-Ib*	*floR*	*dfrA27*	*ARR-3*	
KPN_2021 ST17.1	*bla*$_{NDM-1}$	*bla*$_{CTX-M-14}$、*bla*$_{SHV-96}$、*bla*$_{SHV-94}$、*bla*$_{SHV-172}$		*OqxA*、*OqxB*、*qnrA1*	*tet(A)*、*tet(D)*	*fosA*	*sul1*、*sul2*	*aph(6)-Id*、*aph(3'')-Ib*、*ant(2'')-Ia*		*dfrA1*		
KPN_2021 ST22.1	*bla*$_{NDM-1}$、*bla*$_{OXA-1}$	*bla*$_{SHV-99}$、*bla*$_{KPC-2}$、*bla*$_{CTX-M-15}$、*bla*$_{TEM-1B}$	*mph(A)*	*OqxA*、*OqxB*、*aac(6')-Ib-cr*、*qnrB4*、*qnrB1*	*tet(A)*	*fosA*	*sul1*、*sul2*	*aac(3)-Iia*、*aph(3'')-Ib*、*aac(6')-Ib-cr*、*aadA16*	*catB3*	*dfrA14*、*dfrA27*	*ARR-3*	

Strain	Carbapenems	β-lactams	Macrolides	Quinolones	Tetracycline	Fosfomycin	Sulfonamides	Aminoglycosides	Amphenicols	Trimethoprim	Rifampicin	Polymyxin
KPN_2021 ST628.1	bla_{NDM-1}、bla_{OXA-1}	bla_{SHV-78}、bla_{SHV-26}、$bla_{CTX-M-14}$		*OqxA*、*OqxB*、*aac(6')-Ib-cr*	*tet(A)*	*fosA*、*fosA7*	*sul1*	*aac(3)-Iid*、*aac(6')-Ib-cr*	*catB3*	*dfrA1*	*ARR-3*	
ECO_2017 ST156.1	bla_{NDM-5}、bla_{OXA-10}	$bla_{TEM-186}$、$bla_{CTX-M-65}$、$bla_{TEM-148}$ 、$bla_{TEM-217}$、$bla_{TEM-234}$、$bla_{TEM-207}$等	*mph(A)*	*qnrS1*	*tet(A)*	*fosA3*	*sul2*、*sul1*、*sul3*	*aac(3)-IV*、*aadA1*、*aph(3')-Ia*、*rmtB*、*aadA2b*、*aph(3')-Iia*、*aph(4)-Ia*、*aph(6)-Id*、*aph(3'')-Ib*	*cmlA1*、*floR*	*dfrA14*、*dfrA12*	*ARR-2*	*mcr-1.1*
ECO_2017 ST167.1	bla_{OXA-1}、bla_{NDM-5}	$bla_{CTX-M-15}$、bla_{TEM-1B}	*mph(A)*	*aac(6')-Ib-cr*	*tet(A)*		*sul1*		*catB3*	*dfrA12*		
ECO_2018 ST405.1	bla_{OXA-1}、bla_{NDM-9}	$bla_{CTX-M-55}$	*mph(A)*、*mph(E)*、*msr(E)*	*aac(6')-Ib-cr*	*tet(B)*	*fosA3*	*sul1*		*catB3*	*dfrA17*、*dfrA12*	*ARR-3*	
ECO_2018 ST297.1	bla_{NDM-5}	$bla_{TEM-141}$、$bla_{TEM-214}$、bla_{TEM-1B}、$bla_{TEM-206}$、$bla_{TEM-216}$、$bla_{CTX-M-55}$等			*tet(A)*		*sul2*		*floR*			
ECO_2018 ST167.1	bla_{NDM-5}、bla_{OXA-1}	$bla_{CTX-M-15}$	*mph(A)*	*aac(6')-Ib-cr*	*tet(A)*		*sul1*、*sul2*		*catB3*	*dfrA12*		

Strain	Carbapenems	β-lactams	Macrolides	Quinolones	Tetracycline	Fosfomycin	Sulfonamides	Aminoglycosides	Amphenicols	Trimethoprim	Rifampicin	Polymyxin
ECO_2019 ST410.1	*bla*$_{NDM-5}$、*bla*$_{OXA-1}$	*bla*$_{TEM-1B}$、*bla*$_{CMY-2}$	*mph(A)*	*aac(6')-Ib-cr*	*tet(B)*		*sul1*	*aac(6')-Ib-cr*、*aadA5*、*aac(3)-IId*	*catB3*	*dfrA17*		
ECO_2019 ST167.1	*bla*$_{NDM-7}$	*bla*$_{CTX-M-55}$、*bla*$_{TEM-1B}$			*tet(A)*	*fosA3*	*sul3*	*aph(3')-Ia*、*aadA2b*、*aph(6)-Id*、*aac(3)-IId*、*aadA1*、*aph(3'')-Ib*	*cmlA1*	*dfrA17*		
ECO_2019 ST2309.1	*bla*$_{NDM-5}$	*bla*$_{TEM-1B}$、*bla*$_{CTX-M-65}$	*mph(A)*	*qnrS1*	*tet(A)*		*sul3*、*sul1*	*aadA24*、*aph(3')-Ia*、*aph(3'')-Ib*、*aadA1*、*ant(3'')-Ia*、*aac(3)-IV*、*aph(4)-Ia*、*aadA22*、*aadA2*、*aph(6)-Id*	*cmlA1*、*floR*	*dfrA12*	*ARR-2*	
ECO_2019 ST167.2	*bla*$_{NDM-5}$				*tet(A)*							
ECO_2019 ST410.2	*bla*$_{OXA-1}$、*bla*$_{NDM-5}$	*bla*$_{CTX-M-15}$、*bla*$_{TEM-1B}$ 、*bla*$_{CMY-2}$		*aac(6')-Ib-cr*	*tet(B)*		*sul1*、*sul2*	*aadA5*、*aadA2*、*aph(6)-Id*、*aac(3)-IId*、*aac(6')-Ib-cr*	*catB3*	*dfrA12*、*dfrA17*		
ECO_2020 ST6388.1	*bla*$_{NDM-5}$	*bla*$_{CTX-M-55}$、*bla*$_{TEM-1B}$	*mph(A)*		*tet(A)*	*fosA3*	*sul1*、*sul2*	*aph(3')-Ia*、*aph(6)-Id*、*aadA5*、*aac(3)-IId*、*aph(3'')-Ib*	*catA1*、*floR*	*dfrA17*		

Strain	Carbapenems	β-lactams	Macrolides	Quinolones	Tetracycline	Fosfomycin	Sulfonamides	Aminoglycosides	Amphenicols	Trimethoprim	Rifampicin	Polymyxin
ECO_2020 ST617.1	*bla*$_{NDM-4}$、*bla*$_{OXA-10}$	*bla*$_{TEM-1B}$	*mph(A)*		*tet(A)*		*sul3*、*sul1*	*aph(6)-Id*、*aadA2*、*aph(3')-Ia*、*aadA22*、*aadA1*	*floR*、*cml*	*dfrA14*、*dfrA12*	*ARR-2*	*mcr-1.1*
ECO_2020 ST410.1	*bla*$_{NDM-5}$、*bla*$_{OXA-1}$	*bla*$_{TEM-1B}$、*bla*$_{CMY-2}$、*bla*$_{CTX-M-15}$	*mph(A)*	*aac(6')-Ib-cr*	*tet(B)*		*sul1*、*sul2*	*aadA5*、*aac(6')-Ib-cr*、*aph(3'')-Ib*、*aac(3)-IId*、*aadA2*、*aph(6)-Id*	*catB3*	*dfrA12*、*dfrA17*		
ECO_2020 ST410.2	*bla*$_{OXA-1}$、*bla*$_{NDM-5}$	*bla*$_{CMY-2}$、*bla*$_{TEM-1B}$、*bla*$_{CTX-M-15}$	*mph(A)*	*aac(6')-Ib-cr*	*tet(B)*		*sul1*、*sul2*	*aadA5*、*aph(6)-Id*、*aac(6')-Ib-cr*、*aadA2*、*aac(3)-IId*、*aph(3'')-Ib*	*catB3*	*dfrA12*、*dfrA17*		
ECO_2021 ST167.1	*bla*$_{NDM-5}$	*bla*$_{CTX-M-65}$、*bla*$_{TEM-1B}$			*tet(A)*		*sul1*、*sul2*	*aph(6)-Id*、*aadA2*、*aph(3'')-Ib*、*rmtB*	*floR*	*dfrA12*		
ECO_2021 ST410.1	*bla*$_{NDM-5}$、*bla*$_{OXA-1}$	*bla*$_{CTX-M-15}$、*bla*$_{TEM-1B}$、*bla*$_{CMY-2}$	*mph(A)*	*aac(6')-Ib-cr*	*tet(B)*		*sul1*	*aadA5*、*aac(3)-IId*、*aac(6')-Ib-cr*	*catB3*	*dfrA17*		
